# Koplik's Sign in Measles

**Published:** 1899-06-24

**Authors:** 


### KOPLIK'S SIGN IN MEASLES.

Dr. Henry Koplik,[1] writing on the subject of the spots on the buccal mucous membrane, which he has described as diagnostic of measles, says that it may be stated with absolute confidence that this sign appears in measles only, and never in any other condition whatever, either in health or disease. Of fifty-two consecutive cases examined at his clinic, and subsequently visited at their own homes, the diagnosis of measles was made in thirty-two before any sign of an eruption appeared on the body; in three of these the diagnosis was made three days before any exanthema appeared, in nine the "sign" appeared forty-eight hours before the appearance of the exanthema, and in the remaining twenty cases fully twenty-fours elapsed before any eruption appeared on the skin. In none of the fifty-two cases was the diagnosis wrong. Now the "sign" is an eruption which runs through a certain course. It is at first discrete, then becomes numerous and confluent, and when the skin eruption appears and is spreading it is at its height. It consists of small irregular spots of a bright red colour, in the centre of each spot being a minute whitish-blue speck, which are to be found on the mucous membrane lining the cheeks and the lips. This eruption at first remains discrete, but as the rose-red spots with their bluish-white centres increase in numbers they coalesce, and a peculiar appearance of the mucous membrane results, there being large areas of rose-red studded all over with minute raised bluish-white specks, relieved here and there by the normal hue of the uninvaded mucous membrane. Finally, the whole buccal and labial mucous membrane becomes of a uniform rose-red colour studded with myriads of these bluish-white specks. The chief value of the sign, however, is in the early stage of the disease, as an indication of the onset of measles, showing itself as it does before the occurrence of the eruption, sometimes before conjunctivitis is to be seen, and at a time when little or no fever is present. But Koplik's sign is also of service in diagnosis, and especially in the differentiation of measles from rötheln. In the latter condition there may be a few spots on the fauces and the soft palate, as also there may in scarlet fever, but the characteristic appearance on the buccal and labial mucous membrane is not found.

[1] Med. News, June 3.

### THE RESULTS OF NEEDLE PUNCTURES.

Mr. Jalland, of York, describes in the *Practitioner* some of the evil results which he has seen to follow the apparently simple injury of puncture with a needle. In one of his cases, a needle which penetrated the knee-joint of a dressmaker had the effect of rapidly setting up suppuration within the joint, leading to its entire disorganisation. The pus which was evacuated was intensely fetid, and ultimately amputation had to be performed. This was a case of mere puncture, the needle having been withdrawn immediately. Other instances are given in which the needle remained, and one need hardly say how greatly the X-rays may help in the diagnosis and localisation of the mischief in such cases.

